# A systematic review of factors affecting adherence to malaria chemoprophylaxis amongst travellers from non-endemic countries

**DOI:** 10.1186/s12936-020-3104-4

**Published:** 2020-01-13

**Authors:** Julian Ahluwalia, Samantha K. Brooks, John Weinman, G. James Rubin

**Affiliations:** 1 King’s College London, GKT School of Medical Education, London, UK; 2 Department of Psychological Medicine, King’s College London, Weston Education Centre, Cutcombe Road, London, SE5 9RJ UK; 3 King’s College London, Institute of Pharmaceutical Science, London, UK

**Keywords:** Adherence, Uptake, Chemoprophylaxis, Prevention

## Abstract

**Background:**

The aim of this systematic review was to identify predictors of actual or intended adherence with malaria chemoprophylaxis amongst travellers from non-endemic countries visiting endemic countries.

**Methods:**

A systematic review of the literature was conducted using MEDLINE, Embase, PsycINFO and Global Health databases for studies published up to April 2019. Studies were included if they assessed reasons for adherence among people travelling from a country where malaria was not endemic to a country where it was.

**Results:**

Thirty-two studies were included. Predictors of adherence were categorized as relating to either the nature of the travel or the traveller themselves. The three main predictors associated with nature of travel included: destination (e.g. country visited, urban *vs* rural areas), length of travel and type of travel (e.g. package *vs* backpacking holiday). The four main traveller-associated predictors were: age, reason for travel (e.g. business, leisure or visiting friends and relatives), perceived risk of catching malaria and experienced or expected medication effects.

**Conclusions:**

In order to improve adherence, clinicians should focus on travellers who are least likely to exhibit adherent behaviour. This includes travellers visiting destinations known to have lower adherence figures (such as rural areas), backpackers, business travellers, younger travellers and those travelling for longer periods of time. They should also check to ensure travellers’ perceptions of the risks of malaria are realistic. Where appropriate, misperceptions (such as believing that curing malaria is easier than taking prophylaxis or that travellers visiting relatives have some level of innate immunity) should be corrected. All travellers should be informed of the potential side-effects of medication and given guidance on why it is nonetheless beneficial to continue to take prophylaxis. Further research is required to test interventions to improve adherence.

## Background

Malaria is a disease transmitted by female mosquitos of the genus *Anopheles*, which bite mainly in the evening and at night, and act as the primary vector for spreading the *Plasmodium* protozoa. Of the five parasite species that can cause malaria in humans, the most severe and deadly form of the disease is caused by *Plasmodium falciparum*. It is estimated that across the globe 3.2 billion people are at risk of malaria, making it one of the world’s greatest public health concerns. The World Health Organization (WHO) [1] reported that in 2015 there were over 214 million cases of malaria and 438,000 deaths attributable to the disease. The demographics most at risk of malarial infection include pregnant women, young children and those visiting endemic countries from areas where malaria is not present [1]. Malaria is a notifiable disease in the UK, which has one of the highest rates of imported malaria in Europe [2].

Each year over 125 million international travellers are placed at risk of malaria infection by visiting the 97 countries and territories in the world where malaria is currently endemic [3]. Despite global mortality rates attributable to malaria falling by 60% since 2000 [1], it appears likely to become even more of a burden for some countries, such as Britain, due to both increased travel abroad and immigration from countries where malaria is prevalent. It is estimated that 10,000–30,000 international travellers are affected by contracted malaria every year [4] with possible underreporting meaning that this figure could be higher. Of these cases, 90% of travellers do not develop symptoms until they return home [2].

Those travelling from non-endemic countries are placed at a significantly higher risk of malaria infection and consequences as they typically lack any immunity to malaria. In 2018, there were 1683 imported cases of malaria reported in the UK [5]. Delays in diagnosis, treatment and an increased risk of morbidity are possible for travellers arriving home to countries where clinicians are unfamiliar with malaria [6].

In order to avoid contracting malaria an individual may try to avoid bites (through mosquito nets, for example, or sprays) and using chemoprophylaxis. The use of anti-malarial medication to help prevent travellers from contracting malaria is strongly recommended by guidelines from the National Institute of Health and Clinical Excellence [7], with those visiting at-risk regions advised to take one of several types of tablet 1 to 3 weeks prior to, during, and 2 to 4 weeks after their trip [2]. Despite this, adherence to the full course of malaria prophylaxis medication is often sub-optimal, as shown in this review. Improving adherence may be the key to reducing rates of malaria among travellers and is emphasised in prevention guidance documents [8].

A growing body of research has explored the reasons why people often fail to adhere to medication across a range of contexts. One recent model of adherence specifies the importance of three broad categories of variable, suggesting that capability, motivation and opportunity predict behaviour [9]. Michie, van Stralen and West [10] define someone’s capability as their ‘psychological and physical capacity’ to take part in a given activity; motivation relates to both automatic, habitual processes along with reflective reasoning; and opportunity encompasses all factors outside the individual including both social opportunity afforded by the cultural milieu and physical opportunity. Not all factors relating to adherence fall neatly within this model however, with some (such as forgetting) seeming to straddle categories.

This systematic review sought to identify the range of variables that have been identified as affecting adherence to currently used anti-malarial drugs given as prophylaxis to non-immune adults and children who are travelling to regions with endemic malaria.

## Methods

The review was conducted in accordance with the preferred reporting items for systematic reviews and meta-analyses (PRISMA) guidelines [11].

### Search strategy

A search was performed using Ovid, in the MEDLINE, Embase, PsycINFO and Global Health databases. Databases were searched from inception. The search was initially conducted on 28th December 2015, updated on 28th January 2017 and further updated on 4th April 2019. The following search terms were used: (malaria) AND (adherence OR compliance OR uptake) AND (prophyl* OR prevention OR atovaquone OR proguanil OR malarone OR chloroquine OR doxycycline OR mefloquine OR lariam OR primaquine). The results of this search were then filtered to remove duplicates and non-English results. Any studies categorized on the databases as ‘non-human’ were also removed. Those publications that were left were assessed for their relevance against the inclusion criteria by first screening their titles and abstracts and then screening the full-texts of any that appeared potentially relevant.

### Inclusion criteria

Studies were included if they:

- Presented original data (excluding, for example, review or commentary papers);
- Assessed people travelling from a non-endemic country to an endemic country;
- Assessed a non-military sample;
- Assessed the association between one or more variables and actual or intended adherence with malaria prophylaxis medication, or else described the self-reported reasons given by participants for their actual or intended adherence to malaria prophylaxis;
- Used a quantitative method (excluding purely qualitative studies);
- Were published in English;
- Were published as a full peer-reviewed paper (excluding, for example, conference papers and abstracts).

### Data extraction

For every included paper, details were tabulated relating to citation, year of publication, the sample that was studied, sample size, study design, the adherence rate to chemoprophylaxis, predictors of actual or intended adherence, self-reported reasons for actual or intended adherence, and a quality assessment grade. Any other details felt to be of importance in understanding the study were also noted.

Where possible, adherence rates for each study were calculated as the percentage of all participants who took all their tablets as recommended. It was not always possible to calculate this, however. For example, the nearest data reported by Cunningham et al. [12] was the percentage of participants who took more than 95% of their tablets as recommended.

### Quality assessment

A methodological quality assessment was conducted based on a simplified version of the Delphi list [13]. Studies received one point for meeting each of the following criteria:

1. Eligibility criteria specified (with reasons for exclusion).
2. Large sample (i.e. over 1000 participants).
3. Appropriate statistical analysis and data reporting (such as p values) of significant predictors of adherence.

Studies scoring zero or one out of three were classified as ‘low’ quality. Those scoring two were classified as ‘medium’ quality. Studies scoring three were categorized as ‘high’ quality.

### Procedure

The search, data extraction, assessment of risk of bias and data synthesis was carried out by JA with advice from GJR and JW. An updated search, data extraction, assessment of risk of bias and data synthesis was carried out by SKB with advice from GJR. Any uncertainties were resolved through discussion.

## Results

**Fig. 1:**
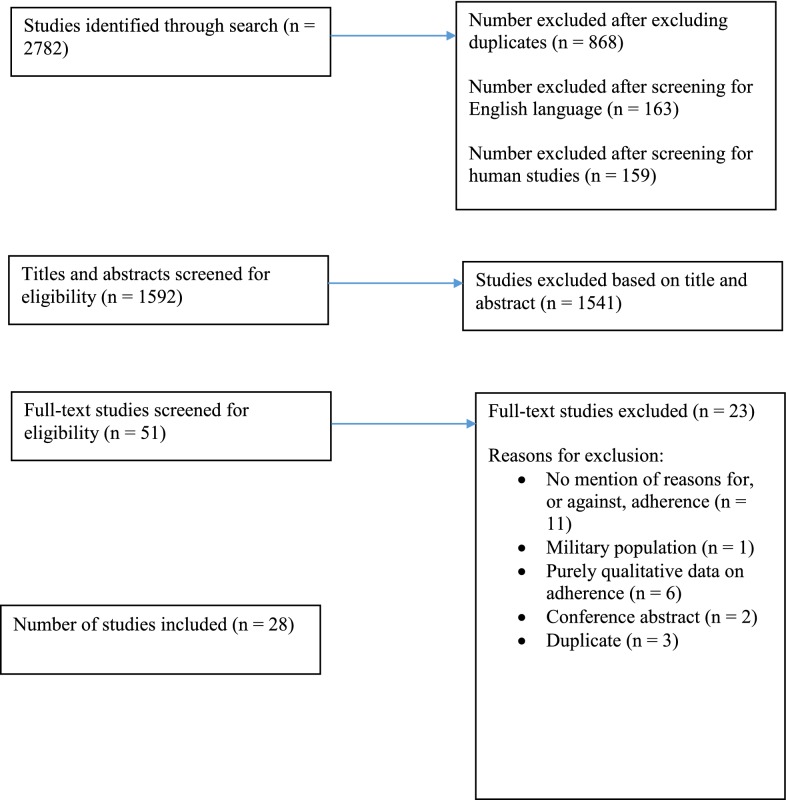
Search Tree (Search conducted on 28th December 2015. Results do not include one additional study identified in an update on 28th January 2017, two additional studies identified in an update on 4th April 2019 nor a study identified during publication review.)

The initial literature search resulted in a total of 2782 citations. After excluding duplicates, as well as non-human studies and studies not in English, 1592 citations were left. After screening of titles and abstracts, 51 papers appeared potentially relevant and were examined in full and 28 publications were included. One additional study was included following the 2017 update, bringing the total to 29. Two additional studies were included following the 2019 update, thus increasing the total to 31. One additional study [14] was identified during publication review, taking the total to 32. Figure 1 shows the results of the initial search.

Included studies were conducted in several countries including the Netherlands, Germany, Canada, USA, UK, Israel and France, and with a wide range of participants including short and long-term travellers, those travelling for pleasure, those traveling for business, Peace Corps volunteers and others. Most studies relied on self-report questionnaires. Most used a cross-sectional design, asking returning travellers to report on their adherence.

Table 1 provides detailed information about the methods of each included study, together with adherence rates, factors associated with actual or intended adherence, and self-reported reasons for actual or intended adherence.

**Table 1:** summary of findings from included studies

Citation	Sample population	Sample size	Study design	Adherence rate to chemoprophylaxis^a^	Predictors of adherence (factors highlighted in *italics* indicate a significant result)	Self-reported reasons for adherence	Quality
Phillips-Howard et al. [15]	British travellers	326	Cohort study	48%		Complex information and receiving conflicting advice when they contacted other advisory services were recorded as reasons for non-adherence, with only 257 travellers felt to have fully understood the information. Forgetting, considering prophylaxis to be unnecessary, advised to stop (by local people or members of their peer group), side-effects and illness, and travelling at short notice so no tablets available	Low
Lobel et al. [16]	US citizens travelling to Haiti or Africa	4042	Cross-sectional survey	42.4%	Prophylaxis use was associated with receiving *pretravel advice* (p < 0.001) (forewarning risk of malaria, chemoprophylaxis recommendation, medical consultation). The source that provided the information was not an influence on chemoprophylaxis use		High
Hilton et al. [17]	US travellers	214	Retrospective telephone interview	81% in patients older than 40, 59% of patients younger than 40	A greater number of travellers older than 40 were fully adherent compared with those under 40 (significance not reported)		Low
Lobel et al. [18]	European and North American travellers to Kenya	5489	Longitudinal study	52% (of 3469 travellers who used chemoprophylaxis)	*Business travellers* (compared with tourists) were significantly more likely to be non-adherent (26%, p < 0.001), as were those *visiting friends and relatives* (32%, p < 0.001) compared with tourists. Logistic regression analysis showed that adherence was particularly poor among people who *visited friends and relatives* (37%), *travelled for more than three weeks* (39%), *experienced adverse reactions* (40%), *used proguanil* (31%), and among *young travellers from the UK* (43%) (P < 0.001)		High
Steffen et al. [19]	European travellers	42,202	Cross-sectional survey	55.4% (mean)	*Weekly medication* (p < 0.001) was significantly associated with increased adherence when compared with daily, twice weekly and thrice weekly medications. Those who *stayed abroad over 3* *months* (p < 0.001) compared with those travelling for less than 3 months were significantly more likely to be non-adherent as were those who had had *several previous journeys* compared to those on their first journey to the tropics (p < 0.001). Severe adverse drug reactions were experienced by 1.5–4.4% of travellers and caused at least 200 of them to prematurely stop their chemoprophylaxis		High
Held et al. [20]	European or North American travellers returning to Berlin	507	Retrospective study	38% (195/507)	Significantly higher adherence was noted amongst: *patients using only one information source compared to those who used no information source* (p = 0.0026); *shorter travel duration* (37.2 ± 38.5 days (mean ± SD) in contrast to 69.8 ± 93.5 days in the group of patients with no adherence p = 0.00001); *older patients* compared to those aged under 55 (20/27 adherent > 54 compared with 175/476 < 55, p = 0.0001); *travel destination* with Southern and East African destinations showing highest levels of adherence (p = 0.0054); *package tours* (p = 0.0001) compared with those who had organised the travel themselves		Medium
Cobelens et al. [21]	Dutch travellers	547	Cross-sectional survey	60%	Adherence was significantly affected by *geographical areas* travelled to (p < 0.001)—for example adherence was 45% in South America compared with 78% in East Africa, those *aged under 29* (p = 0.027) were significantly more likely to be non-adherent compared with those aged over 29, those who had *previous travel* experience (p = 0.031) were significantly more likely to be non-adherent and those with *adventurous travel style* (p < 0.001)—such as backpacking—were also more likely to be non-adherent compared with their non-adventurous counterparts Gender, education and travel purpose were not significantly associated with adherence in a logistic regression model	Self-reported reasons for early discontinuation included believing it unnecessary to continue prophylaxis during part of the journey due to the perceived low risk (43.9%), stopped on advice by others (12.2%), experiencing adverse reactions (11.6%), negligence (17.1%). Other reasons mentioned (all less than 5%) included: lack of awareness of the need to continue, loss of tablets, bad taste of the tablets, insufficient amounts prescribed, fear of developing adverse effects and (possible) pregnancy	Medium
Chatterjee [22]	Travellers to India	452	Cross-sectional survey	71% (320/452)	Females appeared more likely to be non-adherent than males (significance not reported), along with travellers under 30 years old (significance not reported). Travellers on visits lasting longer than 3 weeks tended to be less adherent (significance not reported)	Reasons for poor adherence included: inadequate dose or incorrect drug (21%); pretravel information deficit (45%); active decision (33%); side-effects (25%)	Low
Banerjee et al. [23]	UK GPs travelling to South Asia	145	Telephone survey	46%		Some self-reported reasons for poor adherence included: thinking the area was free from malaria (34%); not wishing to take prophylaxis (18%); experiencing previous side-effects (10%); believing they had long-term immunity (10%); had no time to obtain prophylaxis (4%); costs (2%) and thinking it was easier to cure than to take the medication (2%); travelled for a short period and took the risk (2%)	Low
Lobel et al. [24]	North American and European travellers to East Africa	6633	Cross-sectional study	61.7%	Adherence was lowest in those who used a *daily drug* as opposed to a weekly schedule (OR = 4.03; 95% CI 3.32–4.89), attributed an *adverse event* (OR = 2.23; 95% CI 1.80–2.76) to the prophylaxis, *stayed more than a month* (OR = 3.32; 95% CI 2.64–4.18), those who were *non-tourism* travellers (OR = 3.04; 95% CI 2.42–3.82), those aged *under 40* (OR = 2.19; 95% CI 1.76–2.71)		High
Farquharson et al. [25]	Travellers attending a travel medicine clinic	130	Prospective study using regression analysis	62% full adherence and 25% partial adherence	There were no significant differences across the adherence groups for age, gender, ethnicity, nationality, education, previous travel or previous experience of anti-malarial medication Multiple logistic regression showed that poor adherence (compared to full adherence) was associated with *greater amounts of health professional discussion about adherence in the medical consultation* (OR = 0.7, 95% CI 0.6–0.9). Increased likelihood of full adherence (compared to partial adherence) was associated with *perceived benefits of taking prophylaxis* (OR = 1.4, 95% CI 1.1–1.9), *going for a longer trip* (OR = 3.6, 95% CI 1.5–8.7), and *greater amounts of traveller information and questions in the consultation* (OR = 1.0, 95% CI 0.0–1.1). Poor adherence (compared to partial adherence) was associated with *going for a longer trip* (OR = 0.2, 95% CI 0.1–0.6) and *greater amounts of traveller information and questions* (OR = 1.0, 95% CI 0.9–1.0)		Medium
Jute et al. [26]	Expatriates working on a Mali mine	90	Cross-sectional survey	72%		Some self-reported reasons for poor adherence included concerns over adverse side-effects, presumed immunity from long-term residence in Africa and a high standard of on-site care	Low
Hamer et al. [27]	Expatriate corporate workers in Ghana	42	Cross-sectional survey	0% amongst those based over a year 81% of those based three months or less	*Duration of stay* (p < 0.01) was significantly associated with lowered adherence – for example none of those based over a year were still taking their chemoprophylaxis compared with 81% of those based three months or less	Common reasons for discontinuing malaria prophylaxis include medication side-effects, low perceived malaria risk and suggestions from colleagues on the job site	Medium
Ropers et al. [28]	German travellers to Kenya, Senegal and Thailand	1001	Cross-sectional survey	69% in Kenya 53% in Senegal 6% Thailand	*Travel to Kenya compared to Senegal* resulted in a significantly higher adherence rate to chemoprophylaxis (p = 0.021), *Receiving advice (from either a medical or non-medical professional)* significantly increased adherence when compared with those who received no advice (p < 0.001), *Correct risk perception* (p < 0.001) was associated with a significant increase in prophylaxis adherence. *Increased length of travel* (for example comparing those travelling less than 14 days to those travelling 15–21 days) was associated with increased adherence (p < 0.001)	Reasons for poor adherence included absence of mosquitoes (53%) and ‘adverse effects’ with their medication (22%)	High
Roukens et al. [6]	Non-immune expatriate business travellers	2350	Cross-sectional, web-based study	45%	*Malaria awareness and CMK training* (RR = 2.2; 95% CI 1.6–3.2); *long-term travellers* less likely to be adherent compared to rotators or visitors (p < 0.001)		High
Baggett et al. [29]	US residents travelling to India	1302	Cross-sectional study	VFRs (visiting friends and relatives) 16.3%; non-VFRs 39.4%	Factors significantly associated with lower adherence to chemoprophylaxis included *travelling to India in the previous 5* *years* (POR = 0.46; 95% CI 0.31–0.67) and travelling with the purpose of *VFRs (visiting friends and relatives)* (p < 0.001). Taking chemoprophylaxis was also more common among *US citizens* (POR = 2.71; 95% CI 1.91–3.85)		High
Alon et al. [30]	Israeli travel clinic	394	Telephone interview	60.7% in over 60 age group 33.8% in 20–30 age group	*Elderly travellers* (p < 0.01)—those aged 60 and over—were significantly more likely to be adherent than those in the 20–30 age group		Medium
Depetrillo et al. [31]	Travellers from the United States	104	Prospective, non-blinded study	89%	Factors associated with increased adherence included *travel destination*, with those travelling to regions such as Sub-Saharan Africa having significantly higher adherence levels (p = 0.0063) compared with those travelling for example to Central America. Other significant predictors of non-adherence included *previous travel to a malarious region* (p = 0.0411) compared with those who had never travelled to a malarious region before	Travellers’ self-reported perception of need was felt to be a key influencer in adherence. 7/12 felt it was not necessary, 2/12 were told by their tour guides they did not need to take it and 3/12 reported adverse side-effects	Medium
Dia et al. [32]	French travellers to Senegal	358	Prospective cohort study	71.8%	Factors significantly associated with non-adherence included *reporting at least one gastrointestinal symptom* (p = 0.07) and *non-reporting arthropod bite* (p = 0.04)	The main reasons for not taking medications were: finding it useless (47.1%) and fearing side effects (44.1%)	Medium
Joshi et al. [33]	UK South Asians	400	Cross-sectional survey	49% (1994) and 32% (2004)	Factors associated with an increased adherence with prophylaxis included a *basic knowledge of malaria* (p = 0.003), *perceiving malaria as a critical illness* (p = 0.004) and *defining trip as a holiday* (as opposed, for example, to a ‘visit to friends and family’) (p = 0.043) Age, gender and occupational status did not relate to adherence in either year; years of post-16 education did not relate to adherence in the 2004 sample (not asked in the 1994 survey) Adherence was not related to experience of malaria or having been born in a malarial zone	Reasons given for non-adherence given by partial and zero adherers included: belief in personal immunity (47% in 1994, 43% in 2004); perceived low risk of getting malaria (42% in 1994, 26% in 2004); never heard of tablets (25% in 1994, 27% in 2004; forgot to take/get tablets (21% in 1994, 15% in 2004); dislike taking tablets (14% in 1994, 29% in 2004); believing malaria is easily treatable (9% in 1994, 22% in 2004); local norms (5% in 1994, 17% in 2004)	Medium
Pistone et al. [34]	French adult travellers	13,017 (3066 travellers to malaria-endemic countries)	Retrospective questionnaire study	47.6% in high-risk areas 9.5% in low-risk areas	Factors significantly associated with increased adherence with malaria chemoprophylaxis included *awareness malaria was serious* (OR = 2.03, p = 0.033) and *receiving information from a physician* (OR = 3.01, p = 0.042). When the analysis was reiterated for travellers to low and high-risk areas separately, *older travellers* were less likely to be adherent for the high-risk travellers only (OR = 0.95 for each incremental year of age p = 0.018)		High
Belderok et al. [35]	Dutch short-term travellers	620	Prospective cohort study	75% (466/620) took 100% of recommended tablets	Significant factors associated with adherence included: *travelling to Africa* (OR = 3.5; 95% CI 1.9–6.5) instead of Asia or Latin America; *taking mefloquine* (OR = 5.3; 95% CI 1.2–23.1) compared to atovaquone–proguanil or proguanil; *spending 14–29* *days in endemic areas* (OR = 2.2; 95% CI 1.2–3.8) instead of ≤ 13 days or ≥ 29 days in endemic areas; *concurrent use of DEET for more than 50% of days in high-endemic areas* (OR = 2.6; 95% CI 1.4–4.8)		Medium
Caillet-Gossot et al. [36]	Children under 16 visiting travel medicine centre in Marseille, France	167	Prospective study	66%	*Adherence was significantly higher in those visiting African destinations* (p < 0.02) compared with those taking a trip *to Asia or Indian Ocean* Being aged* < 5* (p < 0.03) was also found to be a predictor of non-adherence as was being from a *mono-parental family* (p < 0.04) Adherence was identical between VFR and tourist children, irrespective of trip duration		Medium
Muller et al. [37]	Adults consulting at a Medical Department for International Travellers’	287	Cross-sectional survey and telephone interview	76.3%	*Travelling to areas of mass tourism (such as Kenya and Senegal)* (p = 0.005) was found to be a predictor of adherence—it was noted that these travellers were also less likely to be seasoned travellers (compared with, for example, long-stay business travellers); *trips shorter than 15* *days* were associated with better adherence (p = 0.001)	Side-effects (20.6%), forgetting (17.6%), too many pills—because of other treatments (17.6%), no mosquitoes seen (13.3%), tiredness (11.8%), did not like taking medication (10.3%), price (2.9%), lack of pills (1.5%)	Medium
Wieten et al. [38]	Travellers to Ghana from the Netherlands	154	Questionnaire survey	53.9% (had started chemoprophylaxis)	Attending pretravel clinic and receiving *pre-travel advice* was related to a greater likelihood of starting chemoprophylaxis (p < 0.01); if a participant *incorrectly thought they had been vaccinated* (p = 0.009) they were also more likely to use chemoprophylaxis *Higher age* (p = 0.004) and travelling for *family purposes* (p = 0.022) rather than business were positively associated with starting chemoprophylaxis. Having *had malaria* (p = 0.028) and spending *more than 6* *weeks in West Africa* (p = 0.001) were negatively associated with starting and buying chemoprophylaxis Those who thought *curing malaria was easier* than taking preventative tablets (p = 0.046) were more likely to be non-adherent—it was felt that subjectively held information is more important than accurate information Previous use of chemoprophylaxis was not found to influence current preventive behaviour		Medium
Cunningham et al. [12]	Foreign and Commonwealth Office employees on long-term placement in endemic areas	327	Questionnaire survey	25.1% had adherence > 95% of prescribed pills 54.4% had adherence < 25% of prescribed pills	*Increasing age* was shown to be significantly associated with improved adherence (Chi squared p < 0.00), *living in an endemic area for more than a year* was significantly correlated with adherence less than 95%, *pregnancy* was associated with lower adherence (87.5% of pregnant women took no prophylaxis) Significant side-effects were reported by 39.5% of respondents and there was a trend between reported side-effects and self-reported adherence < 95% (p = 0.087)	Concerns with long term safety was cited by more than half of individuals with adherence < 25%	Medium
Goldstein et al. [39]	Israelis attending Haifa travel clinics	307	Questionnaire survey	34.7%	*Shorter travel* (p < 0.001), with those who adhered having a travel duration on average 2.6 times shorter than those who did not; travel to *urban areas* (p < 0.01) showed higher adherence; travellers *older than 23* (p = 0.021) showed higher adherence, *backpackers* showed lower levels of adherence (p < 0.01) compared to other travellers		Medium
Landman et al. [40]	Peace Corps volunteers in the Africa region in 2013	781	Questionnaire survey	73%	Factors significantly associated with non-adherence included: being *prescribed mefloquine* (OR = 5.4; 95% CI 3.2–9.0) as opposed to doxycycline or atovaquone–proguanil; if they *were in the peace corps for over a year* (OR = 1.8; 95% CI 1.2–2.8); *being under 26* *years old* (OR = 1.7; 95% CI 1.1–2.6); *not worrying about malaria* (n = 214; OR = 2.6; 95% CI 1.6–4.1); *fears long-term adverse effects* (OR = 1.6; 95% CI 1.1–2.4)	The most common reasons for non-adherence included: forgetting (n = 530, 90%), fear of long-term adverse effects (n = 316, 54%) and experiencing adverse events that volunteers attributed to prophylaxis (n = 297, 51%)	Medium
Shady [41]	Visitors to traveller’s health clinic to obtain malaria prophylaxis	928	Prospective comparative study	81.6% with mefloquine and 79.5% with doxycycline	*University education* (p = 0.005) was a predictor of non-adherence, *travel organized through an agent* showed increased adherence (p = 0.0001) whereas *independently organized travel* (p = 0.0001) was a predictor of non-adherence, *blue-collar workers* (p = 0.0001) showed higher non-adherence compared to white-collar workers Predictors of good adherence for mefloquine group included travel to an *African destination* (p < 0.001), *education above a secondary level* (p < 0.001), *organized travel* (p < 0.05), *travelling for leisure* (p < 0.05) and *Kuwaiti nationality* (p < 0.001) Predictors of good adherence in the doxycycline group included *higher than a secondary level of education* (p < 0.001), *organized travel* (p < 0.001), *travel for leisure* (p < 0.05), travel to an African destination (p = 0.05) and *Kuwaiti nationality* (p < 0.001)		Medium
Stoney et al. [42]	US travellers	370	Cohort study	71.6%	No significant difference for sex (p = 0.74), location of birth (p = 0.49), endemicity of country of birth (p > 0.99), daily vs weekly chemoprophylaxis (p = 0.19), visiting friends or relatives as a reason for travel (p = 0.44), destination as partially or entirely endemic (p = 0.89), or travelling for more or less than 2 weeks (p = 0.19)	Reasons for declining entirely: advised by peers not to take chemoprophylaxis (32%), low perceived risk in area (28%), no mosquitoes present during trip (16%), fear of side effects (16%), cost (8%), had a side effect (4%), unable to fill prescription before trip (4%), other (8%) Reasons for not taking full course: Forgetting (cited by 50% of participants nonadherent during travel), side-effects (31%), not seeing mosquitoes (11%), low perceived risk in area (8%), lost medication (6%), other reason (6%). Data from a post-travel survey, completed by a smaller proportion of participants, are not reported here	Medium
Rolling et al. [43]	German travellers	928	Questionnaire survey	19% carried anti-malarial medication	Neither duration of travel or previous travel experience significantly differed between those carrying anti-malarial medication and those who did not A *medical consultation prior to travelling* was associated with significantly higher odds of carrying anti-malarial medication after adjusting for age, with the highest odds in those having had their *consultation at a travel medicine specialist* (OR 7.83 compared to no consultation)		Medium
Pagès et al. [44]	Malaria cases in Réunion Island (a previously malaria-endemic island; the last indigenous cases were reported in 1967, but international travel has reintroduced the illness)	89	Epidemiological surveillance data; data from Regional Health Agency investigations	29 patients were prescribed chemoprophylaxis: 10 did not buy it, 13 stopped taking it early, 3 took it irregularly, and 5 reported proper adherence. Of the 56 patients not prescribed anti-malarial medication, 24 were not aware they should have consulted a doctor, 21 chose not to, and 11 were not prescribed a medication after their consultation	An absence of chemoprophylaxis *or* poor adherence was found in the majority of malaria cases (96%) regardless of the reason for travel (visiting friends and family vs. other reasons)		Low

^a^Unless otherwise noted, adherence rates reflect percentage of participants taking all tablets as recommended

### Adherence rates

Adherence rates varied widely, ranging from 0% for corporate workers placed in Ghana for over a year [27] to 89% for travellers from the USA [31].

### Self-reported reasons for non-adherence

Forgetting to take the medication was reported as a reason for non-adherence in four studies [15, 33, 37, 40, 42]. Several studies also reported concerns with side-effects: this included concerns about the safety of long-term use of anti-malarial medication [12, 21, 40], as well as experienced (both past and present) or anticipated side-effects [15, 21, 23, 26–28, 31, 33, 37, 40, 42]. Other reasons included having too many pills to take [37]; not seeing any mosquitoes [28, 37, 42]; tiredness [37]; price [23, 37, 42]; lack of pills [37]; not thinking that prophylaxis was necessary [15, 21, 31]; being advised (for example, by a tour guide, locals or colleagues) that it was not necessary [15, 21, 27, 31, 42] and not liking to take medication [33, 37]. A higher perceived risk of catching malaria was associated with greater adherence and having a self-reported low perceived risk [27, 42] was associated with poor adherence. Thinking there was no malaria [23] was associated with poor adherence, as was: presumed immunity [23, 26, 33]; high standard of on-site medical care, for example, at a mine in Mali [26]; taking an active decision not to take medication [22, 23]; being unable to obtain the tablets [23, 42]; thinking it is easier to cure malaria than to take the tablets [23, 33]; having travelled for a short period and deciding to take the risk [23], losing the medication [42] and having travelled at short notice [15].

### Demographic risk factors

Eleven studies found that older participants were more likely to be adherent [12, 17, 18, 20, 21, 24, 30, 32, 38–40]. Another study reported that those under 30 were less likely to be adherent than older people [22], but significance was not reported. In addition, one study showed that those aged under 5 were significantly less likely to take their tablets correctly [36]. However, Joshi et al. [33] found that age was not a predictor of adherence as did Farquharson et al. [25].

A participant’s country of residence was shown to influence adherence in three studies [18, 29, 41], though not in two studies [14, 42]. For example, Baggett et al. [29] showed that US citizens were significantly more likely to be adherent than non-US citizens; whilst Shady [41] found that those of Kuwaiti nationality are shown to have significantly better adherence when compared with non-Kuwaiti individuals.

Socio-economic and education status also influenced adherence rates. Blue-collar workers were more likely to be non-adherent compared with white-collar workers [41]. Having an above secondary-level education improved adherence, whilst going to university was associated with poor adherence [41]. However, three studies [14, 21, 33] found no association between education level and adherence. Children from mono-parental families were more likely to exhibit poor adherence [36]. Cobelens et al. [21] and Farquharson et al. [25] found that gender did not significantly predict adherence; one study [22] reported that females were less likely to be adherent than males, though significance was not reported.

### Travel-related risk factors

Length of stay was reported in numerous studies as influencing adherence, with longer stays associated with poorer adherence [6, 12, 18, 19, 24, 27, 38, 40]. Similarly, shorter travel was shown by most, though not all studies [28, 42], to be associated with better adherence [20, 37, 39]. Farquharson et al. [25] found that poor adherence (compared with partial adherence) was associated with going on a longer trip but also noted that full adherence (compared with partial adherence) was associated with going on a longer trip. Belderok et al. [35] noted that those spending 14 to 29 days in an endemic area were significantly more adherent compared with travellers spending less than 13 days, or more than 29 days, in endemic areas. Conversely, two studies [22, 24] found that poor adherence was associated with travelling for more than 3 weeks, though only one of these studies [24] reported the significance level. One study [43] found that duration of travel was not associated with the likelihood of carrying anti-malarial medication.

Previous travel was shown to be a predictor of poor adherence in four studies [19, 29, 31, 33], though one study found no association between previous travel experience and intention to take anti-malarial medication [43] and a further study [14] also found no significant difference in adherence based on previous travel. Having previously acquired malaria [38] was also shown to be associated with lower rates of adherence, though endemicity of country of birth was not [42].

Destination [20, 21, 28, 31, 35, 36, 41] significantly affected adherence. For example, Depetrillo et al. [31] showed that adherence was greater for those travelling to sub-Saharan Africa compared with central America; Ropers et al. [28] showed there was greater adherence in those travelling to Kenya compared with Senegal; and Shady [41], Belderok et al. [35] and Caillet-Gossot et al. [36] demonstrated that adherence was greatest amongst those travelling to African destinations than to Asia, the Indian Ocean or South America. Travel to urban areas [39] or areas of mass tourism [37] was also associated with better adherence rates that those travelling, for example, to rural areas. No effect was found for whether the destination was partially or entirely endemic [42].

The nature and purpose of travel also significantly affected adherence rates. Package tours [20] and those booked through agents [41] compared with more adventurous (e.g. backpacking [21, 39]) or independent [41] travel styles showed greater adherence rates.

Those travelling to visit friends and relatives, for non-tourism reasons, or those travelling for business, showed worse adherence in most [18, 24, 29, 33], but not all [44, 42] studies—travelling for family-related as opposed to business-related reasons was shown in one study [38] to be associated with greater adherence. Those travelling for a holiday [33] or leisure [41] showed greater adherence than those travelling for business.

### Capability-related risk factors

Having a basic knowledge of malaria [33] or receiving training [6] was associated with better adherence rates, as was receiving pretravel advice [18, 28, 38]. Farquharson et al. [25] noted that poor adherence (compared to full adherence) was associated with greater amounts of health professional discussion in the medical consultation; they also noted that greater amounts of traveller information and questions were associated with both poor adherence (compared with partial adherence) as well as full adherence (compared with partial adherence). There were variations noted depending on the source of the information, however, with higher adherence rates being reported if information was delivered by a physician, compared with if it was not [34]. Those receiving information from one information source were also more likely to be adherent when compared with those using no information source [18]. Rolling et al. [43] found that travellers were more likely to be carrying anti-malarial medication if they had had a medical consultation prior to travelling, especially with a travel medicine specialist. Forgetting to take tablets, which was a self-reported reason for non-adherence, may also be related to capability [15, 33, 37, 40, 42].

### Opportunity-related risk factors

Physical opportunity factors included losing medication or simply not having adequate chemoprophylaxis medication [21, 22]. Social opportunity factors included personal interactions with someone perceived to be knowledgeable about malaria or the side-effects of prophylaxis. Those who received advice from others to discontinue their prophylaxis were less likely to be adherent [15, 21, 27, 31]. Being unable to obtain tablets, which was previously explained as a self-reported reason for non-adherence [23, 42], is also related to opportunity.

### Motivation-related risk factors

Several studies assessed factors relating to motivation. Perhaps the most commonly identified factor was the presence of, or concerns about, side-effects of chemoprophylaxis [18, 19, 24, 32] or concerns about long-term adverse effects of taking the medication [40].

Other motivation related factors included the perceived risk of catching malaria [28, 40], the perceived severity of malaria [33, 34], perceived benefits of prophylaxis [14], perceptions and attitudes towards prophylaxis risks and whether the necessity of taking prophylaxis outweighed these risks (notably the safety and side-effects of any medication taken); or wrongly believing [38] that curing malaria would be easier that taking the prophylaxis. Other incorrect beliefs included perceived immunity ([33]—associated with worse adherence) and false belief in being vaccinated ([38]—associated with better adherence), as well as believing that anti-malarial medications are useless [32]. More automatic motivational factors included habitual behaviours such as previous adherence with prophylaxis recommendations and emotional fear of prophylaxis and side-effects. Farquharson et al. [25] found no significant difference in adherence based on previous experience of anti-malarial medication [14].

Low perceived risk, which was often a self-reported reason for non-adherence—as opposed to greater perceived risk, which was related to greater adherence—is also a motivation-related risk factor [23, 26, 27, 33, 42].

### Medication-related risk factors

Several other predictors were identified pertaining to the medication itself. Weekly medication [19] was shown to be more likely to be taken correctly than daily [24] prophylaxis, though not in all studies [42]. Mefloquine showed better adherence rates as opposed to doxycycline or atovaquone–proguanil [40] and better adherence rates compared with atovaquone–proguanil or proguanil [35]. Lobel et al. [24] found that proguanil was associated with poorer adherence. Concurrent use of DEET [35] was also shown to be associated with better adherence.

Two studies [15, 17] commented that the majority of poor adherence was due to premature cessation of prophylaxis, when the participants returned home to their non-endemic country.

## Discussion

Despite malaria being largely preventable amongst people travelling from non-endemic countries to endemic ones, it is evident that adherence with the recommended full-course of prophylaxis is poor and many studies reveal ‘*a largely inadequate use of malaria chemoprophylaxis*’ [34]. The identified adherence rates among studies identified in this review ranged from 0% for corporate workers placed in Ghana for over a year [27] to 89% for travellers from the USA [31]. Thirteen out of the 32 included papers had adherence rates below 50%. Even amongst those studies which did report higher rates of adherence in certain sub-groups, it was often still possible to identify other sub-groups with poor adherence: for example, although Alon and colleagues reported an adherence rate of 60.7% in their sample of over 60-year-olds, this figure was in stark contrast to the rate of 33.8% reported for their 20–30-year-old sample [30].

Many factors appear to predict adherence. Several of these were repeatedly identified in the literature as important. Destination of travel is one such factor, with travellers to areas in Africa (notably Kenya—[45]) being more likely to adhere to their medication than those traveling to Asia or the Indian Ocean. It is possible that this is due, in part, to travellers perceiving a higher risk in travelling to these countries. Travellers were also more likely to be adherent if visiting urban areas or areas of mass tourism [37], as opposed to rural areas. It is likely that travellers to urban areas and mass tourism areas are also more likely to be inexperienced or travelling for leisure rather than adventure travellers, such as backpackers, and hence hold different attitudes towards prophylaxis.

Age of traveller was shown by many studies to influence adherence rates with older travellers in general having greater adherence rates. The definition of ‘old’ and ‘young’ varied significantly between the studies, however, and more research is needed to understand the factors underlying this.

Similarly, length of travel [27] was identified as a key influencer of adherence, with travellers’ adherence falling the longer they were away. This was of note amongst expatriates and peace corps volunteers. Multiple factors might contribute to this effect, including false beliefs in immunity, side-effects, and fears of adverse effects from long-term medication use. Certainly, experiencing or expecting to experience side-effects [32], was a common factor influencing adherence. In this particular study, *‘individuals who reported at least one gastrointestinal symptom (assigned or not to anti*-*malarials) were more likely to be noncompliant*’.

Encouragingly, education [41], awareness training (e.g. covering ‘*the correct use of the curative medication and the need to seek medical care*’ [6]) and pretravel advice was shown to increase adherence with prophylaxis as was consistent information from more than one source. A key role of information may be generating accurate risk perceptions. For those offering advice to travellers, efforts should be made to identify the travellers’ level of understanding of malaria, the likelihood of contracting it and its severity, and attempt to tailor advice accordingly. Travellers may incorrectly assume that curing malaria is easier than having to take the prophylaxis.

Another key finding of the review was that the reason for travel was a strong predictor of adherence rates. Business travellers [18] were significantly more likely to have low adherence compared to those travelling for leisure. Backpackers/adventure travellers [39] had lower adherence in comparison with those on package tours. Those reporting that they were visiting friends and relatives [33] were also significantly less likely to follow chemoprophylaxis recommendations. Many reasons were put forward for these trends. For example, backpackers may be less likely to follow recommendations because they are less informed about the risks having not received advice from a travel agent, because they have not contracted malaria before and perceive their risk to be low, or because they are younger and less concerned with health risks in general.

The results of this review have implications for clinicians who may be able to improve adherence rates of malaria prophylaxis. Many previous attempts at adherence interventions have been unsuccessful, perhaps due to being developed without a sound theoretical basis, lacking a tailored approach matching interventions and individual determinants of non-adherence, and focusing solely on provision of information [9, 46]. Research has therefore moved on to models focusing on patients’ beliefs, abilities and motivations [9]. One example of this is the Capability, Opportunity and Motivation (COM-B) model of behaviour [10], suggesting that behaviour is influenced by the interaction between capability, opportunity and motivation—and, importantly, that behaviour could be modified by targeting these three factors. Allemann et al. [46] developed a list of modifiable determinants of adherence (such as knowledge, beliefs about capabilities, beliefs about consequences, intentions, and memory, among others) and suggested that such determinants should be assessed and matched to appropriate interventions. It is important that future interventions aimed at improving adherence should be personalized, targeting the causes of non-adherence per individual, and should apply the COM-B model rather than simply providing information to patients.

### Quality of the literature

The quality of this systematic review was limited by the methods of the studies reviewed. In this regard, it is notable that only eight included studies were rated as being high quality. Many of the studies used different methods to assess adherence, and factors influencing adherence, amongst a wide range of participants. The definitions of adherence and the length of recommended treatment also differ greatly from study to study, meaning that a participant defined as adherent in one study might not have been in another. For example, Cunningham et al. [12] defined adherence as taking more than 95% of prescribed tablets, whereas Belderok et al. [35] reported those who took all their prescribed tablets; Rolling et al. [43] explored *intention* to adhere to anti-malarial medication simply by asking travellers at an airport if they were carrying tablets. The reliance of many studies on self-reported measures of adherence was also notable. Self-report has limitations as a method, reflecting participant recall and social desirability as well as genuine adherence. Future studies should explore alternative ways of measuring adherence.

The eight high-quality studies tended to have larger participant samples (ranging from 1001 to 42,202). It is perhaps an issue that many studies in this area use small sample sizes and there is a lack of large-scale randomized controlled trials. Those who reported overall adherence rates of the whole sample reported fairly similar prevalence rates (42.4–61.7%). Four of the eight high-quality studies examined pre-travel knowledge/advice as a determinant of adherence, and all found it a significant predictor, while three explored reason for travel and found that non-tourism reasons for travelling were associated with lower adherence.

### Quality of this review

Positively, the findings of this review seem to broadly correspond with a previous review, published in the form of a poster presentation and letter [14, 47]. However, there are some limitations to the review process. Due to pragmatic considerations, this review was limited to papers published in English. Papers in other languages, or which appeared only in the grey literature, may exist which would have added to the conclusions. Similarly, because the initial search itself was carried out by one individual, human error in the compilation of the literature database cannot be discounted. This may have resulted in some studies being erroneously excluded. Because studies with significant findings are more likely to be published and are usually easier to locate and identify, it is possible that some of the apparent predictors of adherence that were identified may be less robust than it currently appears. It should also be noted that conclusions are inevitably constrained by what currently exists in the literature and there may be scope for other, more imaginative interventions to promote adherence. It is also possible that there are different reasons for non-adherence in different populations and that reviews not focusing solely on travellers from non-endemic countries may uncover other factors associated with non-adherence.

## Conclusions

This review identified several predictors of actual and intended adherence to malaria prophylaxis, ranging from country visited, the length of time travelling, and the purpose of visit, amongst other things. Whilst further research in this area is needed, it is hoped that some of these findings may be taken forward to inform interventions. The results suggest that to improve adherence clinicians should concentrate their attention on those groups identified as least likely to exhibit adherent behaviour. They should ensure that they focus on travellers visiting destinations known to have lower adherence rates (such as rural areas), backpackers, business travellers, younger travellers and those travelling for longer periods of time. They should check to ensure that a traveller’s perceived risk of catching malaria is equivalent to the actual risks of travelling and that they do not, for example, wrongly believe that curing malaria is easier than taking prophylaxis or falsely believe that they have some level of innate immunity because they are visiting relatives. All travellers should be informed of the potential side-effects of medication and given guidance on why it is nonetheless beneficial to continue to take prophylaxis medication.

## Data Availability

Not applicable.

## Abbreviations

PRISMA: preferred reporting items for systematic reviews and meta-analyses
